# PITPNA-AS1 abrogates the inhibition of miR-876-5p on WNT5A to facilitate hepatocellular carcinoma progression

**DOI:** 10.1038/s41419-019-2067-2

**Published:** 2019-11-07

**Authors:** Jianmin Sun, Yubao Zhang, Bing Li, Yuandi Dong, Chengming Sun, Fang Zhang, Li Jin, Dongqin Chen, Wansheng Wang

**Affiliations:** 1 Department of Hepatopancreatobiliary Surgery, Harbin Medical University Cancer Hospital, No. 150 Haping Road, Nangang District, Harbin, 150081 Heilongjiang China; 2 Department of Radiotherapy, Sichuan Cancer Hospital & Institute, Sichuan Cancer Center, School of Medicine, University of Electronic Science and Technology of China, Renmin South Rd 55#, Chengdu, 610041 Sichuan China; 3 Laboratory for Advanced Interdisciplinary Research, the First Affiliated Hospital of Wenzhou Medical University, 2 Fuxue Lane, Wenzhou, 325000 Zhejiang China; 4 Department of Medical Oncology, the Affiliated Cancer Hospital of Nanjing Medical University & Jiangsu Cancer Hospital & Jiangsu Institute of Cancer Research, No. 42 Baiziting Road, Xuanwu District, Nanjing, 210000 Jiangsu China; 5 Department of Medical Oncology, the First Affiliated Hospital of Soochow University, No. 188 Shizi Street, Gusu District, Suzhou, 215006 Jiangsu China; 6 Department of Interventional Radiology, the First Affiliated Hospital of Soochow University, No. 188 Shizi Street, Gusu District, Suzhou, 215006 Jiangsu China

**Keywords:** Cancer, Molecular biology

## Abstract

LncRNA PITPNA-AS1 was a newly identified lncRNA which has never been studied in cancers. Whether PITPNA-AS1 participated in the development of hepatocellular carcinoma (HCC) is obscure. Given the coaction of lncRNAs and miRNAs to carcinogenesis, the purpose of the present research is to inquire how PITPNA-AS1 affects HCC progression. Firstly, PITPNA-AS1 was observed to be heightened in HCC tissues. Then function assays proved that overexpressing or silencing PITPNA-AS1 could manipulate the proliferation and motility of HCC cells. Besides, PITPNA-AS1 was located in the cytoplasm. Among the candidate miRNAs of PITPNA-AS1, miR-876-5p was an obvious target. Moreover, mechanism experiments validated that PITPNA-AS1 modulated WNT5A expression by targeting miR-876-5p. Rescue experiments affirmed that WNT5A silencing rescued the miR-876-5p suppression-induced cellular processes in PITPNA-AS1-silenced Hep3B cells. And in vivo experiments determined that PITPNA-AS1 regulated HCC progression in vivo via miR-876-5p/WNT5A pathway. In conclusion, this work shed lights on the modulatory mechanism of PITPNA-AS1/miR-876-5p/WNT5A axis in HCC, which might be pivotal for exploring effective diagnostic biomarkers and treatment strategies for HCC patients.

## Introduction

Hepatocellular carcinoma (HCC) is reported to be the third cause of cancer-associated death globally^1^. Recently, treatment options for HCC patients and evidence-based approaches for optimizing disease management are increasing^2^. Nevertheless, the outcome is still not satisfying in spite of advances in surgical resection, chemotherapy, radiotherapy, and even liver transplantation. Exactly, HCC patients have poor prognosis due to late diagnosis and high recurrence rate^3^.

The pathogenesis etiology of HCC usually comes down to intricate genetic and epigenetic transformations. Long noncoding RNAs (lncRNAs) are widely classified as a group of transcripts with length above 200 nucleotides (nt) and develop refrained protein coding property^4,5^. Past studies have exposed the involvement and importance of lncRNAs in cancer initiation and progression along with different mechanisms^6–8^. For examples, lncRNA EGFR-AS1 accelerates cell growth and metastasis of renal carcinoma by affecting HuR-stabilized EGFR^9^; lncRNA MEG3 represses cellular proliferation and stimulates apoptosis in prostate cancer^10^; PCGEM1 boosts the tumorigenesis and exacerbation of ovarian cancer by RhoA pathway^11^; and lncRNA GAS5 suppresses cell cycle through binding to YBX1 so as to modulate p21 in stomach cancer^12^. Importantly, reports about the association between lncRNAs and liver cancer have been increasing in recent years^13,14^. Biological role of dysregulated lncRNAs has been unveiled in HCC progress^15,16^. However, we still need to make more efforts to seek out additional biomarkers for HCC.

LncRNA PITPNA antisense RNA 1 (PITPNA-AS1) is located in chromosome 17p13.3 and a never studied RNA in cancers. From the cancer genome atlas (TCGA) database, we unexpectedly discovered the ectopic expression of PITPNA-AS1 in HCC. Besides, the aberrant expression level of PITPNA-AS1 is strongly correlated with overall survival rate, metastasis and TNM stage. These data suggested the possible roles of PITPNA-AS1 as a diagnostic and therapeutic target for HCC therapy.

This study firstly found that that PITPNA-AS1 was heightened in HCC and overexpressing or silencing PITPNA-AS1 could affect the proliferation, apoptosis, migration and EMT process of HCC cells. In addition, PITPNA-AS1 was observed to modulate WNT5A expression by targeting miR-876-5p. Rescue and in vivo experiments affirmed that PITPNA-AS1 regulated cell growth and motility of HCC via miR-876-5p/WNT5A pathway. The expression profile and mechanism of PITPNA-AS1 in HCC was displayed in the current research.

## Results

### PITPNA-AS1 was heightened in HCC tissues

**Fig. 1:**
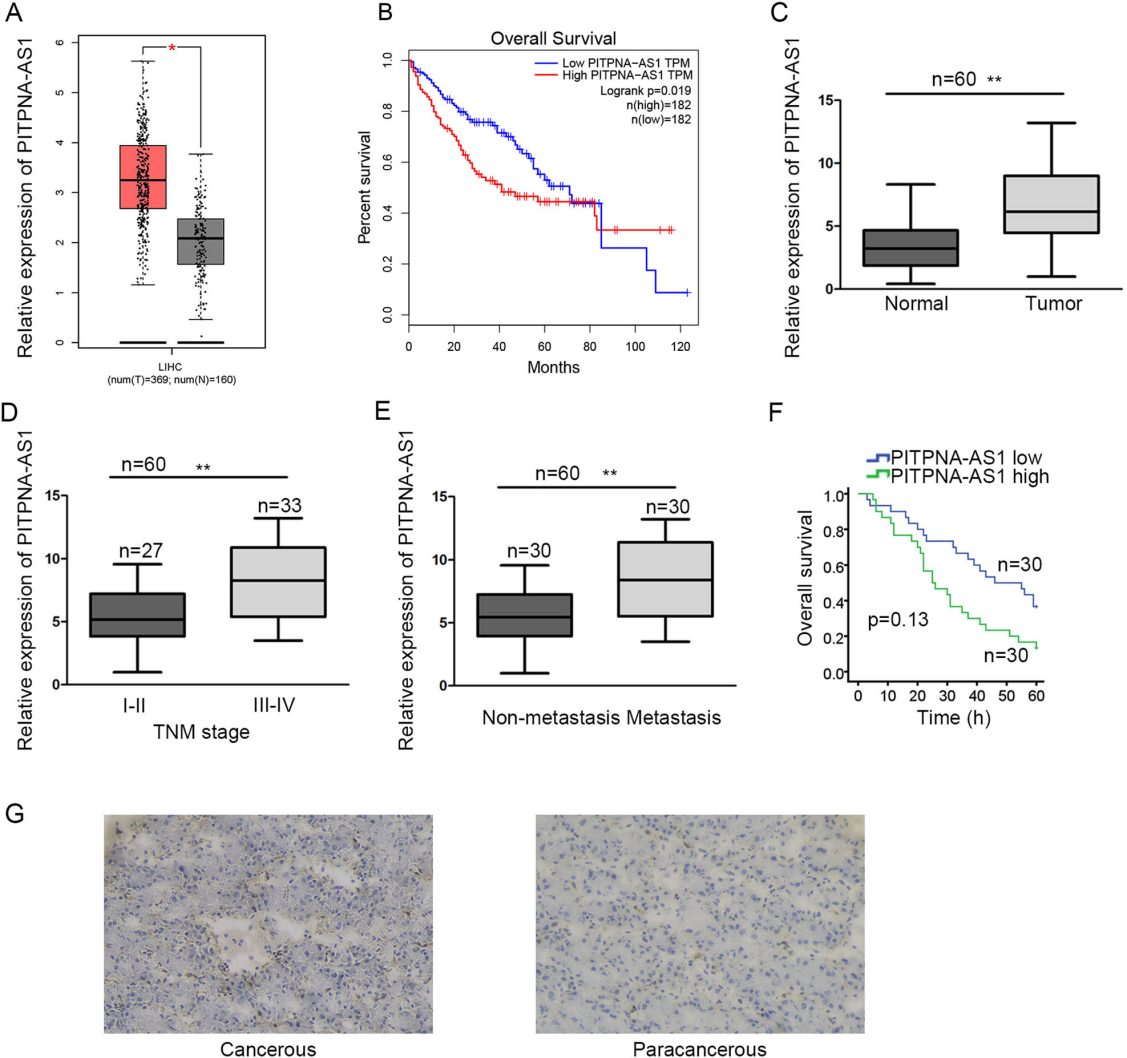
PITPNA-AS1 was heightened in hepatocellular carcinoma tissues. **a** The expression pattern of PITPNA-AS1 in TCGA liver cancer patient samples was obtained from GEPIA. **b** Surviving curve of TCGA liver cancer patients was generated and downloaded from GEPIA in accordance with the median of PITPNA-AS1 expression in tumor samples. **c**–**e** qRT-PCR analysis of PITPNA-AS1 expression in tumor and normal tissues (**c**), tissues in different TNM stages (**d**), and tissues with or without metastasis (**e**). **f** Kaplan–Meier method was utilized to evaluate the survival of 60 HCC patients with high or low level of PITPNA-AS1. **g** Expression pattern of PITPNA-AS1 in cancerous or paracancerous tissues collected from 60 HCC patients was examined with ISH. ^*^P < 0.05, ^**^P < 0.01 indicated statistically significant differences. PITPNA-AS1 phosphatidylinositol transfer protein alpha antisense RNA 1, HCC hepatocellular carcinoma, GEPIA gene expression profiling interactive analysis, TCGA the cancer genome atlas, qRT-PCR quantitative real time polymerase chain reaction, TNM tumor-node metastasis, ISH in situ hybridization

By scanning the TCGA dataset (http://gepia.cancer-pku.cn/index.html), we found PITPNA-AS1, which has never been investigated in cancers, let alone in HCC. Expression of PITPNA-AS1 was obviously higher in tumorous tissues than nontumor samples (Fig. 1a). And PITPNA-AS1 expression was related with the 5-year survival time of HCC patients (Fig. 1b). To probe whether PITPNA-AS1 was implicated in the progression of HCC, we respectively examined PITPNA-AS1 expression in tumorous and normal tissues, tissues in different TNM stages and tissues with or without metastasis. Consistently, PITPNA-AS1 was upregulated in tumorous tissues and tissues with advanced TNM stages or metastasis (Fig. 1c–e). Based on the mean value of PITPNA-AS1 expression, 60 HCC patients were classified into high or low expression group. Using the Kaplan–Meier method, we analyzed and uncovered that patients in high PITPNA-AS1 expression group had worse overall survival time than those with low PITPNA-AS1 expression (Fig. 1f). PITPNA-AS1 expression was further observed in cancerous or paracancerous tissues obtained from HCC patients. As expected, PITPNA-AS1 was expressed higher in cancerous tissues (Fig. 1g). According to the analysis in Table 1, high level of PITPNA-AS1 was significantly associated with metastasis (*P* = 0.019) and TNM stage (*P* = 0.009). These data confirmed that heightened PITPNA-AS1 was related with HCC progression.

**Table 1:** Correlation between PITPNA-AS1 expression and clinical features of hepatocellular carcinoma patients (*n* = 60)

Parameters	PITPNA-AS1 expression		*P* value
	Low	High	
*Age (years)*			
<65	14	17	0.606
≥65	16	13	
*Gender*			
Male	16	19	0.601
Female	14	11	
*Tumor size*			
<5 cm	21	19	0.785
≥5 cm	9	11	
*Metastasis*			
Negative	20	10	0.019^*^
Positive	10	20	
*TNM*			
I–II	19	8	0.009^**^
III–IV	11	22	

Low/high expression was obtained by the sample mean. Pearson *χ*^2^ test. ^*^P < 0.05, ^**^P < 0.01 were considered statistically significant

### Overexpressing or silencing PITPNA-AS1 could promote or inhibit the proliferation ability of HCC cells

**Fig. 2:**
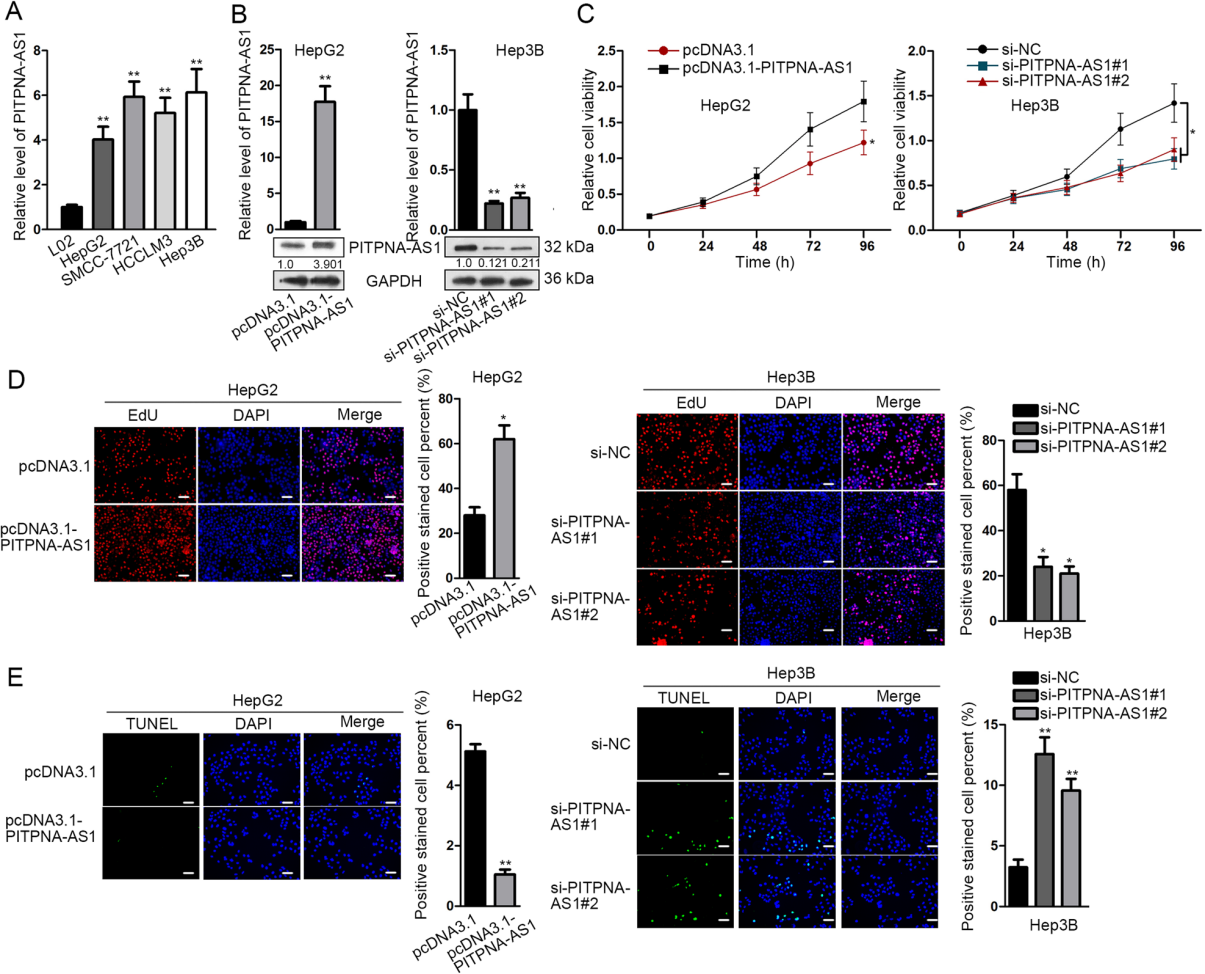
Overexpressing or silencing PITPNA-AS1 could promote or inhibit the proliferation ability of HCC cells. **a** PITPNA-AS1 expression in HCC cells (HepG2, SMCC-7721, HCCLM3, and Hep3B) and normal hepatocytes L02 was tested with qRT-PCR detection. **b** HepG2 cells were transfected with pcDNA3.1-PITPNA-AS1 or pcDNA3.1. Hep3B cells were transfected with si-PITPNA-AS1#1/2/3 or si-NC. The transfection efficacy was testified with qRT-PCR or northern blot assay. **c**, **d** Cell proliferation of transfected HepG2 or Hep3B cells was assessed by CCK-8 and EdU assays. **e** Apoptosis of two transfected cells was examined using TUNEL assay. ^*^P < 0.05, ^**^P < 0.01 indicated statistically significant differences. PITPNA-AS1 phosphatidylinositol transfer protein alpha antisense RNA 1, HCC hepatocellular carcinoma, qRT-PCR quantitative real time polymerase chain reaction, CCK-8 cell counting kit 8, EdU 5-ethynyl-2’-deoxyuridine, TUNEL TdT-mediated DUTP nick end labeling

Then we further inspected the biological function of PITPNA-AS1 in HCC through function experiments. All cultured HCC cell lines including HepG2, SMCC-7721, HCCLM3, and Hep3B presented higher levels of PITPNA-AS1 than normal hepatocyte line L02 (Fig. 2a). After affirming the expression of PITPNA-AS1 in HCC cells, we overexpressed PITPNA-AS1 in HepG2 cells by transfecting pcDNA3.1-PITPNA-AS1 and suppressed PITPNA-AS1 in Hep3B cells by transfecting si-PITPNA-AS1#1/2/3. The transfection efficiency was measured through qRT-PCR and northern blot (Fig. 2b). CCK-8 assay demonstrated that upregulation or silence of PITPNA-AS1 boosted or repressed cell proliferation (Fig. 2c). EdU experiment illustrated the same results (Fig. 2d). And as for cell apoptosis evaluated in TUNEL assay, it indicated that the apoptosis was refrained or stimulated by pcDNA3.1-PITPNA-AS1 or si-PITPNA-AS1#1/2 (Fig. 2e and Supplementary Fig. 1D). Above mentioned function assays were also performed in HCCLM3 cells. PITPNA-AS1 was silenced with siRNAs before functional assays (Supplementary Fig. 2A). Similarly, silence of PITPNA-AS1 hampered proliferation but accelerated apoptosis in HCCLM3 cells (Supplementary Fig. 2B–D). These findings confirmed that PITPNA-AS1 promotion or inhibition facilitated or restrained the proliferation in HCC.

### Overexpressing or silencing PITPNA-AS1 could promote or inhibit the migration and EMT process of HCC cells

**Fig. 3:**
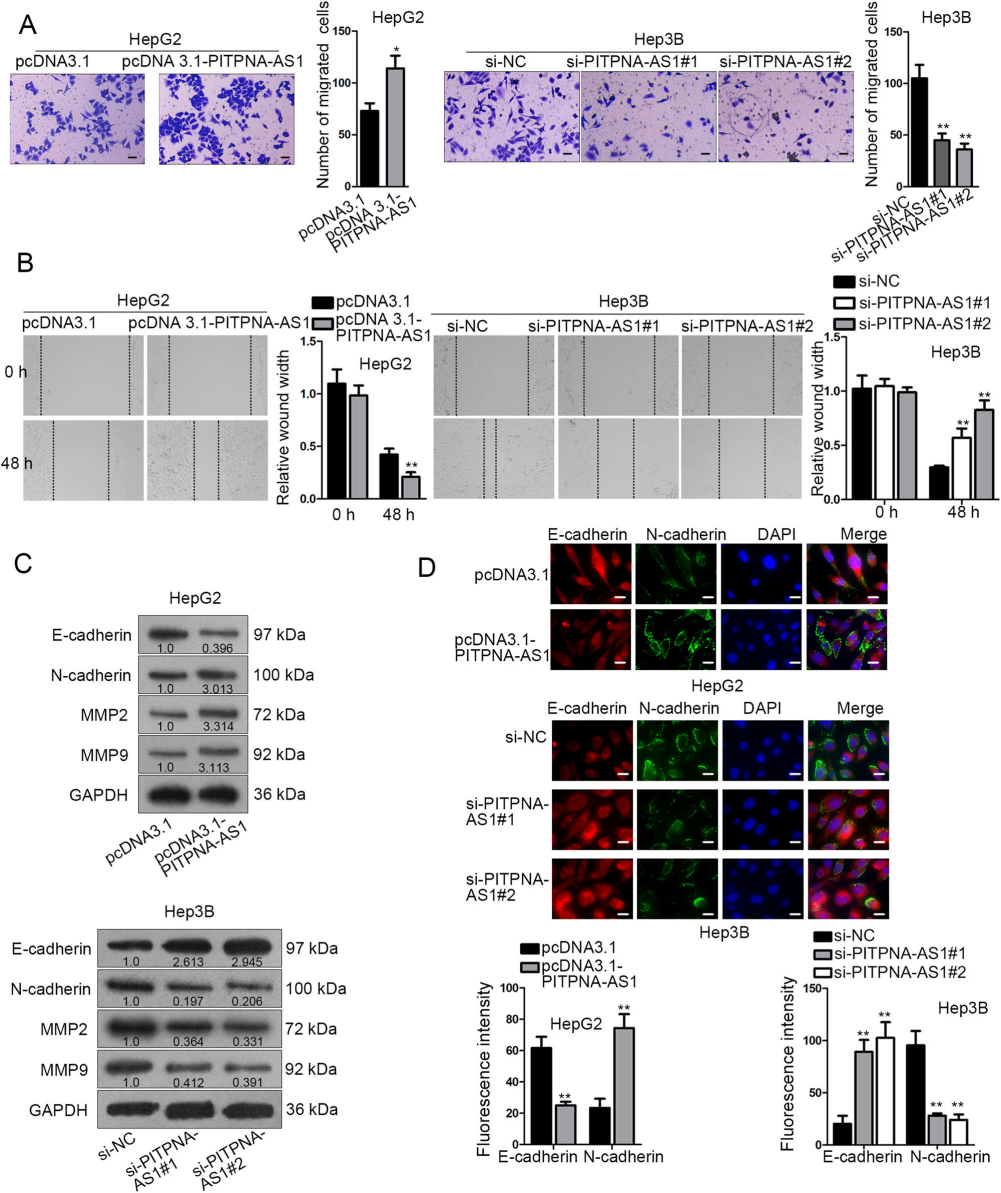
Overexpressing or silencing PITPNA-AS1 could promote or inhibit the migration and EMT process of HCC cells. **a** Transwell assay was conducted to measure the migratory ability. **b** Wound healing assay was carried out in HepG2 and Hep3B cells after indicated transfections. **c** Immunofluorescence (IF) was employed for the impact of PITPNA-AS1 upregulation or downregulation on the expression of EMT markers (E-cadherin and N-cadherin). **d** Western blot analysis of the levels of E-cadherin, N-cadherin, MMP2, and MMP9 in two transfected cells. ^*^P < 0.05, ^**^P < 0.01 indicated statistically significant differences. PITPNA-AS1 phosphatidylinositol transfer protein alpha antisense RNA 1, HCC hepatocellular carcinoma, IF immunofluorescence, EMT epithelial–mesenchymal transition, MMP2 matrix metalloprotein, MMP9 matrix metalloprotein 9

Subsequently, the impact of PITPNA-AS1 on cell motility was estimated. Transwell assay elucidated that cell migration was accelerated by PITPNA-AS1 stimulation, but suppressed by PITPNA-AS1 knockdown (Fig. 3a). Wound healing assay was also performed in transfected HCC cells to assess the impact of PITPNA-AS1 on cell migration. Consistently, PITPNA-AS1 expression had a positive correlation with migratory ability of HCC cells (Fig. 3b). EMT process is acknowledged to be closely associated with migration. Depletion of E-cadherin and increase of N-cadherin are the main characteristic of EMT process. Thus, we evaluated the effect of PITPNA-AS1 on EMT process by detecting relative expression of E-cadherin and N-cadherin in transfected HCC cells with IF assay. As a result, E-cadherin or N-cadherin expression was increased or decreased due to PITPNA-AS1 promotion, and vice versa (Fig. 3c). In western blot, the levels of E-cadherin were lowered and N-cadherin, MMP2 and MMP9 levels were augmented by pcDNA3.1-PITPNA-AS1, and vice versa (Fig. 3d). Similarly, loss-of-function assays were repeated in HCCLM3 cells. Unsurprisingly, we observed that cell migration and EMT process were depleted after downregulation of PITPNA-AS1 (Supplementary Fig. 2E–H). In brief, PITPNA-AS1 upregulation or downregulation facilitated or restrained the motility in HCC.

### MiR-876-5p was significantly targeted by PITPNA-AS1

**Fig. 4:**
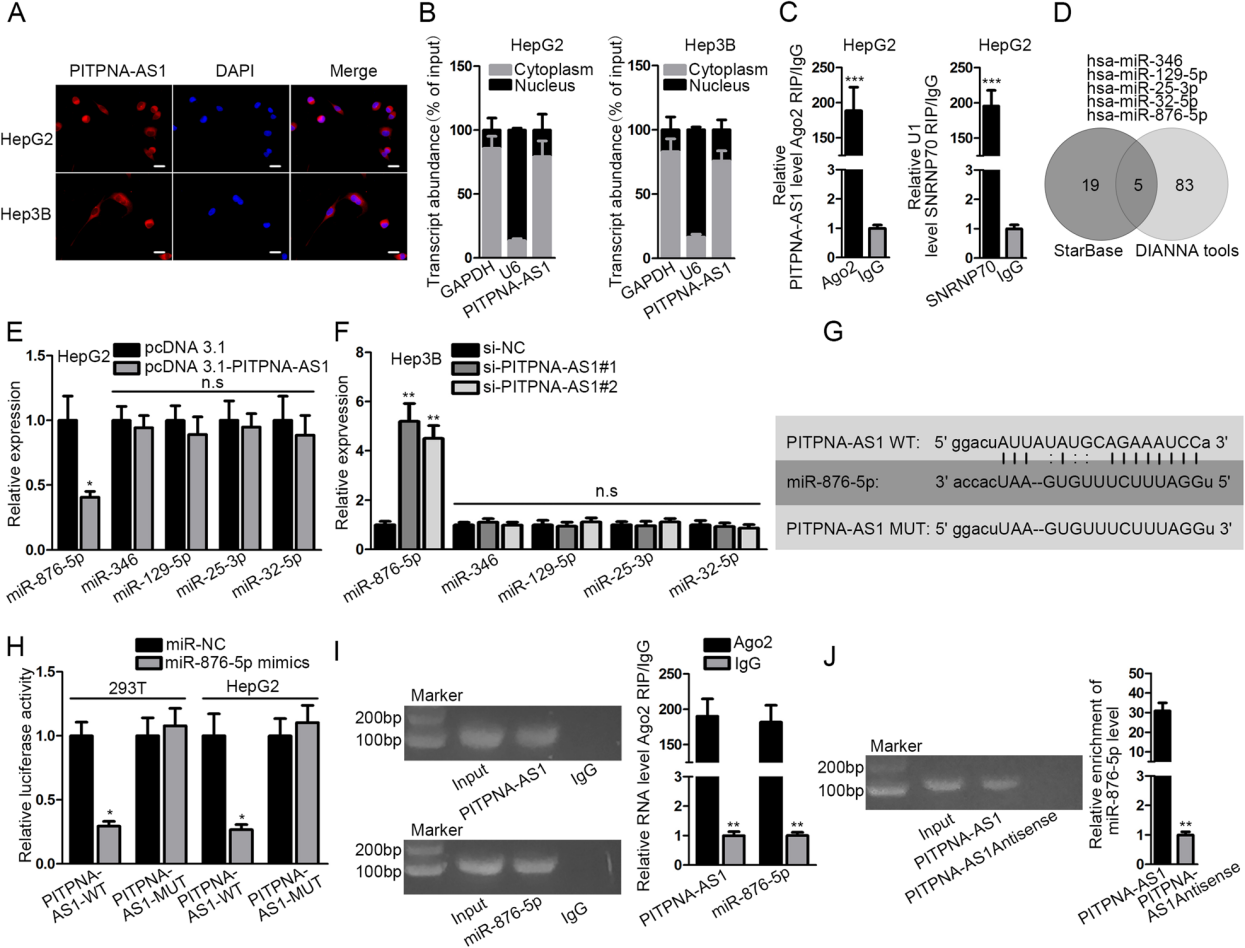
MiR-876-5p was significantly targeted by PITPNA-AS1. **a**, **b** The location of PITPNA-AS1 in HCC cells was tested by FISH and subcellular fractionation assays. **c** The involvement of PITPNA-AS1 in RISC was assessed by Ago2-RIP assay. **d** Five miRNAs that could bind with PITPNA-AS1 were predicted using starBase and DIANA tools. **e**, **f** The levels of five miRNAs were analyzed with qRT-PCR in two HCC cells after PITPNA-AS1 overexpression or silencing. **g** The binding sites between PITPNA-AS1 (wild-type or mutant) and miR-876-6p were exhibited. **h** Luciferase activity of PITPNA-AS1-WT or PITPNA-AS1-MUT was assessed in 293T or HepG2 cells transfected with miR-876-5p mimics or miR-NC. **i** The enrichment of PITPNA-AS1 and miR-876-5p in RISC was identified with Ago2-RIP assay. **j** The interaction between PITPNA-AS1 and miR-876-5p was determined by RNA pull-down assays. ^*^P < 0.05, ^**^P < 0.01, ^***^P < 0.001 indicated statistically significant differences. n.s no significance, PITPNA-AS1 phosphatidylinositol transfer protein alpha antisense RNA 1, HCC hepatocellular carcinoma, RISC RNA-induced silencing complex, qRT-PCR quantitative real time polymerase chain reaction

LncRNAs are reported to exert their roles in the cytoplasm of tumor cells via involving in ceRNA crosstalk^17–19^. To probe the position of PITPNA-AS1 in HCC cells, we performed fluorescent in situ hybridization (FISH) and subcellular fractionation experiments. The results disclosed that PITPNA-AS1 was chiefly located in the cytoplasm (Fig. 4a, b). In addition, RIP experiment unveiled that PITPNA-AS1 was abundantly precipitated by anti-Ago2, suggesting its participation in RISC (Fig. 4c). Through starBase and DIANA tools, we obtained the five common miRNA targets of PITPNA-AS1 which contained hsa-miR-346, hsa-miR-129-5p, hsa-miR-25-3p, hsa-miR-32-5p, and hsa-miR-876-5p. The Venn diagram was inhibited in Fig. 4d. And only miR-876-5p was distinctly silenced when PITPNA-AS1 was upregulated or overtly elevated when PITPNA-AS1 was knocked down (Fig. 4e, f). Then, miR-876-5p was upregulated in 293T and HepG2 cells with miRNA mimics (Supplementary Fig. 3A, left). For luciferase reporter assay, the wild-type and mutant binding sequences were constructed (Fig. 4g). The results uncovered that merely the luciferase activity of PITPNA-AS1-WT was lessened by miR-876-5p mimics (Fig. 4h). In RIP assay, both PITPNA-AS1 and miR-876-5p were enriched in the compound precipitated by anti-Ago2 (Fig. 4i). In RNA pull-down experiment, miR-876-5p was pulled down by biotinylated PITPNA-AS1 but not biotinylated PITPNA-AS1 antisense (Fig. 4j). To sum up, PITPNA-AS1 interacted with miR-876-5p.

### PITPNA-AS1 modulated WNT5A expression by targeting miR-876-5p

**Fig. 5:**
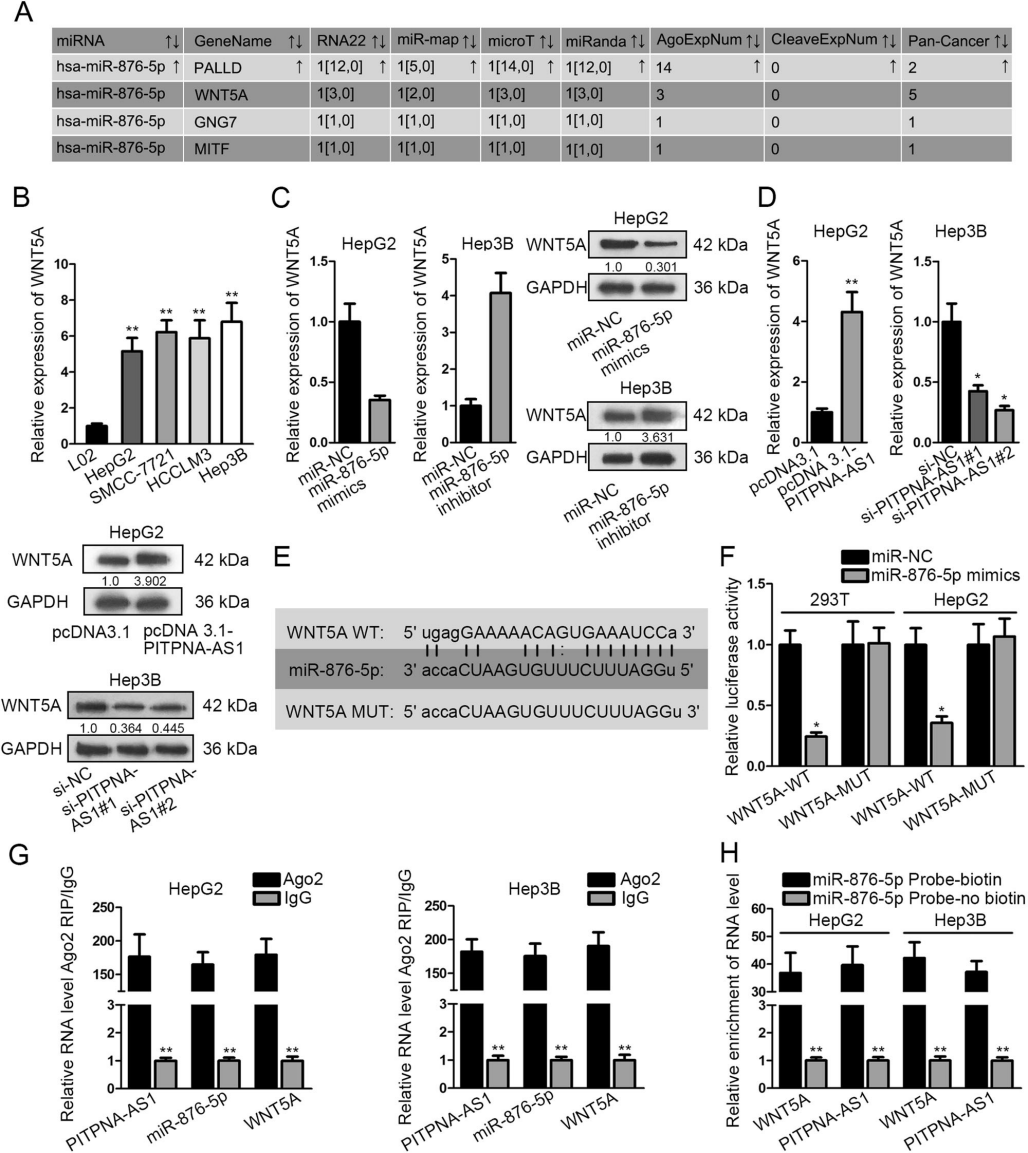
PITPNA-AS1 modulated WNT5A by targeting miR-876-5p. **a** The four predicted targets of miR-876-5p were obtained from five online bioinformatics databases. **b** WNT5A levels in HCC cells and normal hepatocytes were detected with qRT-PCR. **c** The effect of miR-876-5p mimics/inhibitor on WNT5A expression was estimated utilizing qRT-PCR and western blot. **d** WNT5A expression was estimated in HCC cells by means of qRT-PCR and western blot when PITPNA-AS1 was overexpressed or knocked down. **e** The binding sequences between WNT5A (wild-type or mutant) and miR-876-5p were shown. **f** The binding of miR-876-5p to WNT5A was certified by luciferase reporter assay. **g** Identification of the enrichment of PITNA-AS1, miR-876-5p, and WNT5A in RISC with Ago2-RIP assay. **h** RNA pull-down assays was used to confirm the interaction between miR-876-5p and PITNA-AS1 or WNT5A. ^*^P < 0.05, ^**^P < 0.01 indicated statistically significant differences. PITPNA-AS1 phosphatidylinositol transfer protein alpha antisense RNA 1, HCC hepatocellular carcinoma, RISC RNA-induced silencing complex, qRT-PCR quantitative real time polymerase chain reaction, WNT5A Wnt family member 5A

We continued to search for the downstream gene in the regulation mechanism of miR-876-5p. Through the prediction from diverse dataBases (PITA, miRmap, microT, PicTar, and Targetscan), we found four targets of miR-876-5p (Fig. 5a). The lifted WNT5A expression was viewed in HCC cells while that of PALLD, GNG7, and MITF was not found (Fig. 5b and Supplementary Fig. 1A–C). Subsequently, the mRNA and protein levels of WNT5A were separately tested under the transfection of miR-876-5p mimics/inhibitor, pcDNA 3.1-PITPNA-AS1 or si-PITPNA-AS1#1/2 in HCC cells. Transfection efficiency of miR-876-5p inhibitor was detected and identified (Supplementary Fig. 3A, right). WNT5A mRNA and protein expression was repressed by miR-876-5p mimics or si-PITPNA-AS1#1/2 and fortified by miR-876-5p inhibitor or pcDNA 3.1-PITPNA-AS1 (Fig. 5c, d). The wild-type and mutant binding sites of WNT5A for miR-876-5p were presented in Fig. 5e. The luciferase activity of WNT5A-WT was lessened by miR-876-5p mimics while that of WNT5A-MUT varies not (Fig. 5f). Mechanism experiments were employed for the interaction among PITPNA-AS1, miR-876-5p and WNT5A. In RNA pull-down experiment, PITPNA-AS1 and WNT5A were merely precipitated by bio-miR-876-5p probe (Fig. 5g). In RIP assay, PITPNA-AS1, miR-876-5p, and WNT5A co-existed in Ago2 group (Fig. 5h). Collectively, PITPNA-AS1 regulated WNT5A expression via targeting miR-876-5p.

### WNT5A silencing rescued the miR-876-5p suppression-induced cellular processes in PITPNA-AS1-silenced Hep3B cells

**Fig. 6:**
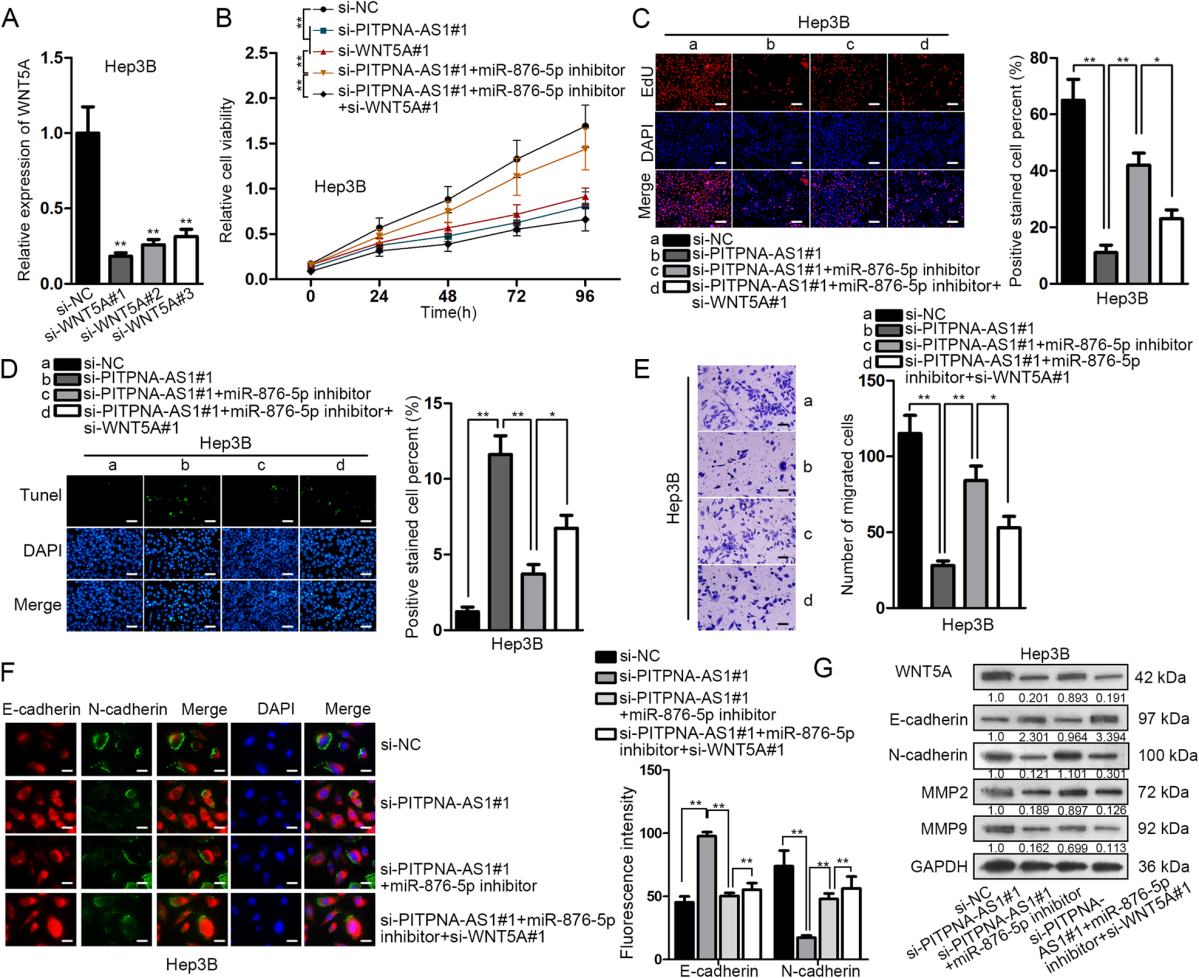
WNT5A silencing rescued the miR-876-5p suppression-induced cellular processes in PITPNA-AS1-silenced Hep3B cells. **a** Knockdown efficacy of WNT5A in Hep3B cells were measured by using qRT-PCR. **b** Cell viability was detected in Hep3B cells transfected with si-NC, si-PITPNA-AS1#1, si-WNT5A#1, si-PITPNA-AS1#1 + miR-876-5p inhibitor and si-PITPNA-AS1#1 + miR-876-5p inhibitor + si-WNT5A#1. Results were obtained using CCK-8 assay. For rescue assays, Hep3B cells were transfected with si-NC, si-PITPNA-AS1#1, si-PITPNA-AS1#1 + miR-876-5p inhibitor and si-PITPNA-AS1#1 + miR-876-5p inhibitor + si-WNT5A#1. **c** Cell proliferation in four groups was detected by EdU assay. **d** TUNEL assay was used to determine the apoptosis ability in four groups. **e** Cell migration was evaluated via transwell assay. **f** IF assay detected E-cadherin and N-cadherin levels in four groups. **g** The levels of WNT5A, E-cadherin, N-cadherin, MMP2, and MMP9 in different groups were examined using western blot assay. ^*^P < 0.05, ^**^P < 0.01 indicated statistically significant differences. PITPNA-AS1 phosphatidylinositol transfer protein alpha antisense RNA 1, HCC hepatocellular carcinoma, WNT5A Wnt family member 5A, CCK-8 cell counting kit 8, EdU 5-ethynyl-2’-deoxyuridine, TUNEL TdT-mediated DUTP nick end labeling, IF immunofluorescence, MMP2 matrix metalloprotein, MMP9 matrix metalloprotein 9

Finally, rescue experiments were carried out. The si-RNAs specifically targeting WNT5A (si-WNT5A#1/2/3) were infected into Hep3B and HCCLM3 cells, resulting in the knockdown of WNT5A (Fig. 6a and Supplementary Fig. 3B). CCK-8 and EdU assays uncovered that the inhibited cell proliferation by si-PITPNA-AS1#1 was promoted by miR-876-5p inhibitor, whereas further abrogated by si-WNT5A#1 (Fig. 6b, c and Supplementary Fig. 3C, D). In TUNEL assay, the induction of si-PITPNA-AS1#1 on the apoptosis was impaired by miR-876-5p inhibitor but recovered by si-WNT5A#1 (Fig. 6d and Supplementary Fig. 3E). Transwell assays observed that cell migration restrained by PITPNA-AS1 silence was boosted by miR-876-5p restraint, which was later partly abolished by WNT5A downregulation (Fig. 6e and Supplementary Fig. 3F). Moreover, the repressive impact of PITPNA-AS1 knockdown on EMT process was restored through miR-876-5p suppression, and the phenomenon was neutralized again through WNT5A prohibition (Fig. 6f). Results of western blot assay were consistent with IF assay (Fig. 6g and Supplementary Fig. 3G). In addition, the protein level of WNT5A decreased by silencing PITPNA-AS1 was recovered by inhibition of miR-876-5p, while this tendency was further reversed after silencing of WNT5A (Fig. 6g). Taken together, PITPNA-AS1 regulated the progression of HCC through miR-876-5p/WNT5A pathway.

### PITPNA-AS1 regulated the progression of HCC in vivo via miR-876-5p/WNT5A pathway

**Fig. 7:**
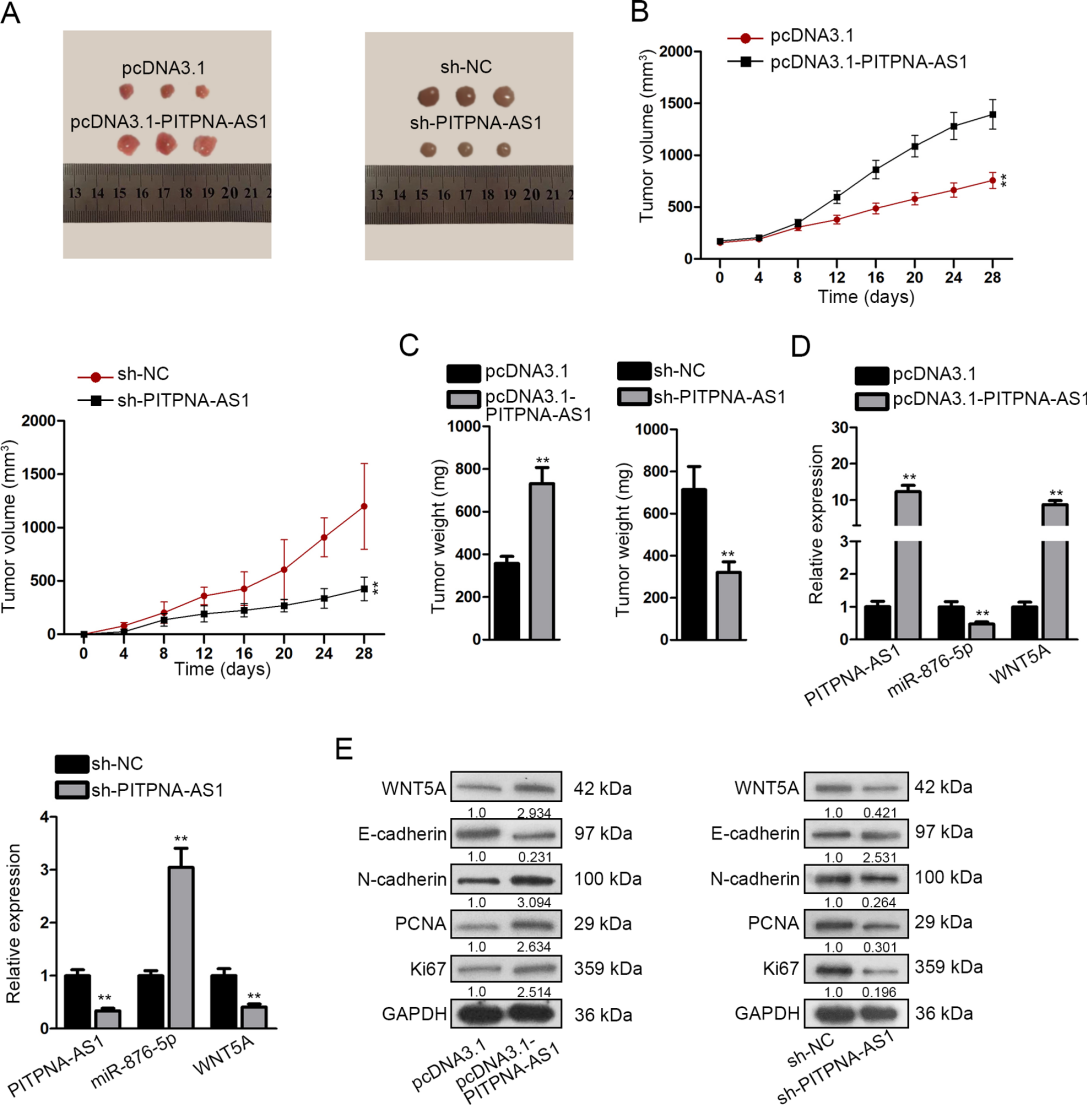
PITPNA-AS1 regulated the progression of HCC in vivo via miR-876-5p/WNT5A pathway. **a** Images of tumors dissected from nude mice that were transplanted with PITPNA-AS1-overexpressed HepG2 cells or PITPNA-AS1-silenced Hep3B cells. **b**, **c** Tumor volume and tumor weight in two groups were quantified. **d** Expression levels of PITPNA-AS1, miR-876-5p, and WNT5A under transfection of pcDNA3.1-PITPNA-AS1 or sh-PITPNA-AS1 were determined by qRT-PCR. **e** Protein level of WNT5A, E-cadherin, N-cadherin, PCNA, and Ki67 was evaluated with western blot assay after stable overexpression or silencing of PITPNA-AS1. ^**^P < 0.01 indicated statistically significant differences. PITPNA-AS1 phosphatidylinositol transfer protein alpha antisense RNA 1, HCC hepatocellular carcinoma, WNT5A Wnt family member 5A, qRT-PCR quantitative real time polymerase chain reaction, PCNA proliferating cell nuclear antigen

To directly assess whether PITPNA-AS1 regulated cellular processes of HCC via miR-876-5p/WNT5A pathway in vivo, nude mice were subcutaneously inoculated with stably transfected HepG2 or Hep3B cells. Images of tumors in different groups were taken (Fig. 7a). Tumor volume and tumor weight were respectively measured and analyzed. PITPNA-AS1 overexpression enhanced the volume and weight of tumors, whereas the opposite results were observed after silencing of PITPNA-AS1 (Fig. 7b, c). Then we evaluated the expression of three genes and found out that PITPNA-AS1 and WNT5A expression was lifted while miR-876-5p expression was silenced due to PITPNA-AS1 upregulation (Fig. 7d). Then, we obtained the opposite results in response to the silencing of PITPNA-AS1. Finally, western blotting examined the levels of WNT5A, EMT-related proteins (E-cadherin and N-cadherin), PCNA and Ki67. Results indicated the reduced levels of E-cadherin and the augmented levels of WNT5A, N-cadherin, PCNA, and Ki67 under PITPNA-AS1 promotion (Fig. 7e). In contrast to PITPNA-AS1 overexpression, stable silence of PITPNA-AS1 led to the promoted level of E-cadherin but the low level of WNT5A, N-cadherin, PCNA and Ki67. Finally, rescue assays were also conducted in vivo to certify the role of PITPNA-AS1/miR-876-5p/WNT5A axis in HCC tumor growth. It was uncovered that the effects of silenced PITPNA-AS1 on tumor growth and protein levels were abolished after co-transfection with miR-876-5p inhibitor, while the function of miR-876-5p was further attenuated by the silencing of WNT5A (Supplementary Fig. 4A–D). We concluded that PITPNA-AS1 regulated HCC progression in vivo via miR-876-5p/WNT5A pathway.

## Discussion

Increasing evidence has disclosed that lncRNAs play a necessary part in human tumors with aberrant levels, including HCC^20–22^. LncRNA DDX11-AS1 epigenetically inhibits LATS2 via binding with EZH2 and DNMT1 in HCC^23^. FOXM1-regulated LINC-ROR affects HCC cell proliferation and Sorafenib-sensitivity^24^. LncRNA TPTEP1 obstructs HCC progression through suppressing STAT3 phosphorylation^25^. Therefore, searching for extra lncRNAs and comprehending their mechanisms are indispensable for the diagnosis and therapy of carcinomas.

The study on the biological role of lncRNA PITPNA antisense RNA 1 (PITPNA-AS1) has neither been researched in HCC nor in other tumors. In consistent with the predicted data that PITPNA-AS1 was high in HCC tissues and linked with the overall survival rate of HCC patients, this study found that PITPNA-AS1 expression was overtly ascended in HCC tissues with metastasis or TNM stage. Prognosis of HCC patients may be affected by various factors, such as Alcohol consumption and Hepatitis B&C. Alteration of gene expression is also correlated with the overall survival of patients. Our present study unveiled the prognostic potential of PITPNA-AS1 in HCC patients. Moreover, PITPNA-AS1 was also upregulated in HCC cells. Gain-of-function and loss-of-function investigations found the oncogenic role of PITPNA-AS1 in promoting HCC growth and metastasis in vitro. It was the first time that PITPNA-AS1 was investigated in HCC, founding the basis for further exploration.

Then we further inspected the potential mechanism underlying PITPNA-AS1. FISH and subcellular fractionation experiments determined the location of PITPNA-AS1 in the cytoplasm, and RIP experiment unveiled the existence of PITPNA-AS1 in RISC, both hinting the feasible participation of PITPNA-AS1 in ceRNA network, a well-known regulatory mechanism that lncRNAs rely on refs. ^26–28^. Here, miR-876-5p, one obviously influenced target of PITPNA-AS1 through starBase and DIANA tools, was recognized for in-depth study. The anti-tumor role of miR-876-5p has been documented yet. For instances, microRNA-876-5p restrains osteosarcoma cell proliferation, migration and invasion through targeting c-Met^29^; microRNA-876-5p represses EMT process and tumor metastasis in HCC via downregulating BCL6 corepressor like 1^30^; miR-876-5p directly targets vimentin to modulate metastasis and invasion of head and neck squamous cell carcinoma^31^. In addition, miR-876-5p took part in the ceRNA model with circular RNA ciRS-7 and MAGE-A family in ESCC^32^. In the current study, mechanism assays unveiled that PITPNA-AS1 targeted miR-876-5p.

Next, we continued to look for the downstream gene. Wnt family member 5A (WNT5A) is implicated in oncogenesis and several developmental processes. According to previous studies, WNT5A promote multiple cellular processes of cancers^33–36^. Accordingly, it was confirmed by our paper that PITPNA-AS1 modulated WNT5A expression by targeting miR-876-5p. The relationship among PITPNA-AS1, miR-876-5p and WNT5A in ceRNA network was firstly presented. Rescue assays validated that WNT5A silencing reversed the miR-876-5p suppression-induced cellular processes in PITPNA-AS1-silenced Hep3B cells, meaning that PITPNA-AS1 regulated HCC progression through miR-876-5p/WNT5A pathway. And the consistent results were also uncovered by in vivo experiments.

Totally, this work expounded the regulatory mechanism underlying PITPNA-AS1 in HCC, providing evidence of efficient diagnostic biomarkers and therapeutic targets of HCC.

## Materials and methods

### Clinical tissue samples

Totally, 60 pairs of HCC and adjacent normal tissues gathered from emrolled patients at Harbin medical university cancer hospital were immediately snap-frozen in liquid nitrogen at −80 °C before use. The tissue samples were divided by TNM stage and metastasis. All patients agreed to sign the informed consent after being informed of the usage for research purposes and they had never received any preoperative chemotherapy or radiotherapy. The usage of tissues in this study was authorized by the ethics committee of Harbin medical university cancer hospital

### Cell lines and cell culture

HCC cells containing HepG2, SMMC-7721, HCCLM3, and Hep3B, normal hepatocyte line L02 and human embryonic kidney (HEK) 293T cells were gained from the Shanghai Cell Bank of Chinese Academy of Science (Shanghai, China). These cells were cultivated in DMEM medium (Invitrogen, Carlsbad, CA, USA) containing 10% FBS (Gibco, Grand Island, NY, USA) in a moist environment of 5% CO_2_ at 37 °C.

### Cell transfection

To enhance miR-876-5p level, miR-876-6p mimics and negative control (miR-NC) synthesized by GenePharma (Shanghai, China) were utilized. MiR-876-5p inhibitors and NC inhibitors were synthesized from the same corporation for silence of miR-876-5p. The sequences were shown as follows: miR-876-5p mimics: UGGAUUUCUUUGUGAAUCACCA; miR-NC: UAAAUUUCUUUGUGAAUCACCA; miR-876-5p inhibitor: UGGUGAUUCACAAAGAAAUCCA; NC inhibitor: UGGUGAGAAAUUUAUUCACAAA. The PITPNA-AS1 expression plasmid (pcDNA3.1-PITPNA-AS1) and specific siRNAs against PITPNA-AS1 (si-PITPNA-AS1#1/2/3) were bought from RiboBio (Guangzhou, China). So was it with WNT5A silencing (si-WNT5A#1/2/3). Herein, pcDNA3.1 and si-NC function as negative controls. Short hairpin RNAs (shRNA) specifically targeting PITPNA-AS1 and control shRNA were constructed by RiboBio to stably downregulate PITPNA-AS1. The transfection was implemented in HCC cells by utilizing Lipofectamine® 2000 (Invitrogen; Thermo Fisher Scientific, Inc., Carlsbad, CA, USA) following the manufacturers’ advice.

### Quantitative real-time PCR (qRT-PCR)

TRIzol reagent (1 ml for per 1 × 10^6^ cells) was prepared for the extraction of total RNA following the normative reference. Post the dissolution, the reverse transcription of RNA (2.5 µl) into cDNA was conducted using Revert AidTM First Strand cDNA Synthesis Kit together with specific primers and reverse transcriptase. The cDNA was subsequently amplified using a Revert AidTM Frist Strand cDNA kit (Fermentas). Threshold cycle (Ct value) of each sample was recorded and analyzed utilizing the 2^−^^ΔΔCt^ method. Data were normalized to GAPDH (for lncRNA and mRNA) or U6 (for miRNA). The sequence is shown as follows: PITPNA-AS1: 5′-GCAGGGTGGATAAAGAGGA-3′ (forward) and 5′-CCTACTGACAGGATGTCCT-3′ (reverse); miR-876-5p: 5′-ACUUAAUGAAACAUUUGGUGGU-3′ (forward) and 5’-TGUUTTUCTTTGTUUUCCUCCU-3′ (reverse); miR-346: 5′-TGTCTGCCCGCATGCC-3′ (forward) and 5′-GAACATGTCTGCGTATCTC-3′ (reverse); miR-129-5p: 5′-ACACTCCTTTTTGCGTCTGGGCTTGC-3′ (forward) and 5′-TGGTGTCGTGGAGTCG-3′ (reverse); miR-25-3p: 5′-CTGGTAGGCATTGCACTTGTCT-3′ (forward) and 5′-TCAACTGGTGTCGTGGAG-3′ (reverse); miR-32-5p: 5′-CGGTATTGCACATTACTAAGTTGCA-3′ (forward) and 5′-CTCGCTTCGGCAGCACA-3′ (reverse); WNT5A: 5′-TCGTTAGCAGCATCAGTCCACA-3′ (forward) and 5′-GACCTGTGCCTTCGTGCCTA-3′ (reverse); PALLD: 5′-CAGGCTGTCAACCAAAGAGGTC-3′ (forward) and 5′-TCGTCTCCACTGTCCCTTGATC-3′ (reverse); GNG7: 5′-CAAAGCGGCGTCTGACCTCATG-3′ (forward) and 5′-GGTTTCTTGTCCTTAAAGGGGTTC-3′ (reverse); MITF: 5′-GGCTTGATGGATCCTGCTTTGC-3′ (forward) and 5′-GAAGGTTGGCTGGACAGGAGTT-3′ (reverse); GAPDH: 5′-GAAGGTGAAGGTCGGAGT-3′ (forward) and 5′-GAAGATGGTGATGGGATTTC-3′ (reverse); U6: 5′-CTCGCTTCGGCAGCACA-3′ (forward), 5′-AACGCTTCACGAATTTGCGT-3′ (reverse).

### Northern blot

Transfection efficiency of PITPNA-AS1 overexpression or knockdown was also determined using Northern blot as previously elucidated^37^.

### Cell counting Kit-8 (CCK-8) assay

HepG2 and Hep3B cells were cultured to the exponential phase, followed by incubation in 96-pore plates for whole night in an incubator at 37 °C which contained 5% CO_2_ at the indicated time of 24, 48, 72, and 96 h. After that, 10 µl of CCK8 reagent was employed for extra 4-h incubation. The absorbance (OD value) at 450 nm was evaluated with a microplate spectrophotometer.

### EdU assay

A Cell-Light EdU DNA Cell Proliferation Kit (RiboBio, Guangzhou, China) was adopted for EdU assay. A total of 3 × 10^4^ cells were inoculated into each well of 24-pore plates and cultivated for nearly 48 h. After being grown with 300 μl EdU for about 2 h, cells were fastened in 4% paraformaldehyde (500 μl; Solarbio) and subsequently stained utilizing Apollo Dye Solution. DAPI was applied to stain nucleic acid and the needed images were acquired under an inverted fluorescence microscope (Carl Zeiss, Jena, Germany). Finally, EdU-positive cells were calculated.

### TUNEL assay

Cells were placed in the 96-well culture dishes (5 × 10^3^ cells/well) for adhesion all night. Cells were rinsed thrice by 0.01 M PBS and fixed for 20 min in 4% paraformaldehyde. Thereafter cells were rinsed thrice utilizing 0.01 M PBS. After that, cells were permeabilized on the ice for 2 min via sodium citrate solution with 0.1% Triton X-100, and again washed with 0.01 M PBS for two times. The prepared TUNEL reaction mixture of 50 µl TdT with 450 µl fluorescein-labelled dUTP solution was supplemented to the dishes (50 µl per well) for 1-h culture in the darkness at 37 °C. After rinsing, each well was added with 50 µl of DAPI reaction solution (5 µg/ml) and incubated for merely 5 min at ambient temperature. The final images and the TUNEL- and DAPI-labelled cells were separately obtained and counted via adopting Thermo Cell Insight CX5.

### Transwell assay

For the exploration of cell migration, transwell assay was conducted. Transwell inserts (8.0 µm pore size; Corning Incorporated, Corning, NY, USA) were employed. HCC cells were plated into the upper chambers with serum-free medium after 2 days of transfection. Meanwhile, the lower chambers were supplemented with medium including 10% FBS to serve as chemoattractant. Post 48 h of incubation of 5% CO_2_ at 37 °C, motionless cells in upper chambers were scraped by cotton swabs, whereas migrated cells to bottom chambers were immobilized (10% methanol, 15 min, 37 °C) and dyed (0.1% crystal violet, 10 min, 37 °C), followed by the detection of cell migration applying an inverted microscope (Olympus, Tokyo, Japan) from about five random fields.

### Wound healing assay

Migratory ability of HCC cells was further evaluated with wound healing assay in accordance with a previous report^38^.

### In vivo tumor growth assay

Based on a previous report^39^, tumor growth in mice was performed. In brief, 1 × 10^7^ stably transfected HepG2 and Hep3B cells were transplanted into the body of 6-week-old BALB/c nude mice (Slac Laboratory Animal Center, Shanghai, China) via subcutaneous injection. Twenty-eight days later, tumors were removed from mice in different groups. Tumor size was measured and calculated as follows: tumor volume was measured at different time point. Tumor weight was calculated after dissection. Permission of conducting animal study was obtained from the Research Ethics Committee of Harbin medical university cancer hospital.

### Fluorescent in situ hybridization (FISH)

A pipette was utilized to transfer the cell suspension liquid onto autoclaved glass slides. Post prehybridization (1× PBS/0.5% Triton X-100), cells were hybridized in hybridization buffer (4% formamide, 10% Dextran sulfate, 1 mg/ml sheared salmon sperm DNA, 1× Denhardt’s solution, 10 mM DDT, 2× SSC and 1 mg/ml yeast transfer RNA) together with FITC-labeled PITPNA-AS1 probes (Geneseed; Guangzhou, China) at 60 °C for whole night. Nuclei were stained using 4, 6-diamidino-2-phenylindole (DAPI) and the corresponding images were visualized relying on a Laser Scanning Confocal Microscope (Leica TCS SP2) (Leica Microsystems, Mannheim, Germany).

### Subcellular fractionation

RNA in the cytoplasm or nucleus was separated, extracted and purified with the help of the Cytoplasmic & Nuclear RNA Purification Kit (Norgen, Belmont, CA) abiding by the supplier’s suggestions. The expression of U6, GAPDH and PITPNA-AS1 were separately evaluated through qRT-PCR.

### Immunofluorescence (IF)

Transfected HCC cells were douched thrice using 0.01 M PBS, fixed by adopting 4% paraformaldehyde for one quarter at indoor temperature, and washed for three times by 0.01 M PBS. For the purpose of blocking nonspecific binding and background, 5% goat serum with 0.5% Triton X-100 was added and incubated for half an hour at indoor temperature. Next, the primary antibody anti-E-cadherin or anti-N-cadherin (Proteintech) was added to the plates in a wet box and cultivated all night at 4 °C. Subsequent to the washing with 0.01 M PBS, the secondary antibody conjugated by Alexa488 fluorescein (Invitrogen) was replenished and incubated in the darkness for 1 h at 37 °C. The cell nuclei were dyed via DAPI (2 µg/ml) at regular temperature for transient 10 min. In the end, the images were viewed and visualized with the help of an immunofluorescence microscope.

### Luciferase reporter assay

Wild-type or mutant PITPNA-AS1 3′-UTR sequences possessing target sites for miR-876-5p were respectively constructed into the pGL3 vector (Promega, Madison, WI, USA) to produce the wild PITPNA-AS1 (PITPNA-AS1-WT) or mutant PITPNA-AS1 (PITPNA-AS1-MUT) luciferase reporter. Similarly, the wild-type WNT5A (WNT5A-WT) or mutant WNT5A (WNT5A-MUT) luciferase reporter was established. Then HCC cells were co-transfected with miR-876-5p mimics or miR-NC and PITPNA-AS1-WT/MUT or WNT5A-WT/MUT by Lipofectamine^™^ 2000 (Invitrogen). 48 h post the transfection, the Dual Luciferase Reporter Assay kit (Promega) was employed to measure the relative luciferase signals.

### RNA immunoprecipitation (RIP) assay

The interaction among studied genes was confirmed by RIP assay utilizing a Magna RIP RNA Binding Protein Immunoprecipitation Kit (Bersinbio, Guangzhou, China) as the manufacturer advised. 293T and HepG2 cells (2 × 10^7^) were lysed with complete RIP lysis buffer to generate the cell lysates, which were later separated into two equal parts and cultured with 5 μg antibody against Argonaute2 (Ago2; Millipore), anti-SNRNP70 (Millipore) or nonspecific IgG (Millipore) along with rotation at 4 °C overnight. Thereafter, magnetic beads were supplemented into the cell lysates for 1 h of incubation at 4 °C, followed by extra 1 h of incubation with Proteinase K at 55 °C. RNA Extraction Reagent (Solarbio) was employed to gain the enriched RNA for subsequent qRT-PCR detection of the expression of the researched genes involving U1, PITPNA-AS1, miR-876-5p, and WNT5A.

### RNA pull-down

For the interplay between PITPNA-AS1 and miR-876-5p, cells were transfected with biotinylated PITPNA-AS1 sense, PITPNA-AS1 antisense or negative control (bio-NC) (Guangzhou RiboBio Co., Ltd.). AS for the association among the three main genes, biotinylated miR-876-5p was used to pull down PITPNA-AS1 and WNT5A, with non-biotinylated miR-876-5p as negative control. Two days after transfection, cell lysates were isolated and then grown with Dynabeads M-280 Streptavidin (Invitrogen, CA, USA) for 3 h at 4 °Cobeying the franchiser’s protocol. The beads were washed thrice using lysis buffer and once using high salt buffer (pH 8.0 and 500 mM NaCl, 1% Triton X-100, 0.1% SDS, 20 mM Tris-HCl and 2 mMEDTA). The obtained RNAs were purified utilizing TRIzol in the qRT-PCR analysis.

### Western blot

Total protein was harvested utilizing RIPA lysis buffer (98%; Beyotime) and cocktail pill (2%; Roche) and the supernatant was obtained by centrifuging the lysates. Then the BCA Protein Assay Kit (Beyotime) was applied to determine the protein concentration. Following the mixture with 1× sodium dodecyl sulfate polyacrylamide gel electrophoresis (SDS-PAGE) protein sample buffer (Biosharp), proteins were divided by SDS-PAGE (10%). Subsequently, it was transferred to the polyvinylidene fluoride (PVDF) membranes. After that, the PVDF membranes were transiently blocked by 5% defatted milk in 1× tris-buffered saline (pH 7.6, 50 mM Tris and 150 mM NaCl) for about 2 h at regular temperature. Next, the membranes were probed at 4 °C overnight with 5% BSA buffer which contained primary antibodies against E-cadherin, N-cadherin, MMP2, and MMP9 (Bioss), and WNT5A, PCNA, Ki67, and GAPDH (Abbkine), and cultured with horseradish peroxidase-labeled goat anti mouse secondary antibody (Abbkine). GAPDH acted as the internal reference. ImageJ software (NIH) was for quantifying the final band intensity.

### Bioinformatics analysis

Expression pattern of PITPNA-AS1 in TCGA liver cancer samples and its association with patients’ survival were acquired through GEPIA online database (http://gepia.cancer-pku.cn/index.html). Through starBase (http://www.sysu.edu.cn/403.html) and DIANA tools (http://carolina.imis.athena-innovation.gr/diana_tools/web/index.php), we obtained the five common miRNA targets of PITPNA-AS1. Four targets of miR-876-5p were obtained through the prediction from five dataBases (PITA, miRmap, microT, PicTar, and Targetscan).

### Statistical analysis

All data of triplicated assays were analyzed using SPSS 16.0 software (IBM Corporation, Armonk, NY, USA) and represented as mean ± SD. Kaplan–Meier method was used to analyze the potential effect of PITPNA-AS1 on the overall survival of 60 HCC patients. Differences which compared two groups were analyzed via Student's *t* test, while differences comparing among three groups were evaluated with one-way ANOVA. Results of CCK-8 was analyzed with two-way ANOVA. As long as *P* < 0.05, the difference was considered to be statistically significant.

## Supplementary information

Supplementary Figure legends

Supplementary Figure 1

Supplementary Figure 2

Supplementary Figure 3

Supplementary Figure 4

Supplementary Table 1
