# Supplementary material for: PITPNA-AS1 abrogates the inhibition of miR-876-5p on WNT5A to facilitate hepatocellular carcinoma progression
Source: Cell Death Dis. 2019 Nov 7;10(11):844. doi: 10.1038/s41419-019-2067-2 (PMC6838072; doi:10.1038/s41419-019-2067-2)
Supplement: Supplementary file 1 — Supplementary Figure legends [file 41419_2019_2067_MOESM1_ESM.docx]

**Supplementary Figure 1** (A-C) The expression levels of PALLD, GNG7 and MITF in HCC cells and normal hepatocytes, as dissected by qRT-PCR. (D) Replication of TUNEL assay conducted in HepG2 cells transfected with pcDNA3.1 or pcDNA-PITPNA-AS1. ^**^P<0.01 indicated statistically significant differences. n.s: no significance. PITPNA-AS1: phosphatidylinositol transfer protein alpha antisense RNA 1; HCC: hepatocellular carcinoma; qRT-PCR: quantitative real time polymerase chain reaction; TUNEL: dT-mediated DUTP nick end labeling; PALLD: palladin, cytoskeletal associated protein; GNG7: G protein subunit gamma 7; MITF: melanocyte inducing transcription factor.

**Supplementary Figure 2** (A) HCCLM3 cells were transfected with si-PITPNA-AS1#1/2/3 or si-NC. The transfection efficacy was testified with qRT-PCR. (B-C) Cell proliferation of transfected HCCLM3 cells was assessed by CCK-8 and EdU assays. (D) Apoptosis of two transfected cells was examined using TUNEL assay. (E) Transwell assay was conducted to measure the migratory ability of PITPNA-AS1-downregulated HCCLM3 cell. (F) Wound healing assay was carried out in HCCLM3 cells after silence of PITPNA-AS1. (G) Immunofluorescence (IF) was employed for the impact of PITPNA-AS1 downregulation on the expression of EMT markers (E-cadherin and N-cadherin). (H) Western blot analysis of the levels of E-cadherin, N-cadherin, MMP2 and MMP9 in two transfected cells. ^**^P<0.01 indicated statistically significant differences. PITPNA-AS1: phosphatidylinositol transfer protein alpha antisense RNA 1; qRT-PCR: quantitative real time polymerase chain reaction; CCK-8: cell counting kit 8; EdU: 5-ethynyl-2’-deoxyuridine; TUNEL: TdT-mediated DUTP nick end labeling; IF: immunofluorescence; MMP2: matrix metalloprotein; MMP9: matrix metalloprotein 9.

**Supplementary Figure 3** Rescue assays were conducted in HCCLM3 cells transfected with si-NC, si-PITPNA-AS1#1, si-PITPNA-AS1#1 + miR-876-5p inhibitor and si-PITPNA-AS1#1 + miR-876-5p inhibitor + si-WNT5A#1. (A) Upregulation of miR-876-5p in 293T and HepG2 cells was conducted using miR-876-5p mimics. MiR-876-5p suppression was identified in Hep3B cells transfected with corresponding miRNA inhibitors. (B) Knockdown efficacy of WNT5A in HCCLM3 cells were determined by using qRT-PCR. (C-D) Cell proliferation in four groups was detected by CCK-8 and EdU assays. (E) TUNEL assay was used to determine the apoptosis ability in four groups. (F) Cell migration was evaluated via transwell assay. (G) The levels of WNT5A, E-cadherin, N-cadherin, MMP2 and MMP9 in different groups were examined using western blot assay. ^**^P<0.01 indicated statistically significant differences. PITPNA-AS1: phosphatidylinositol transfer protein alpha antisense RNA 1; HCC: hepatocellular carcinoma; qRT-PCR: quantitative real time polymerase chain reaction; CCK-8: cell counting kit 8; EdU: 5-ethynyl-2’-deoxyuridine; TUNEL: TdT-mediated DUTP nick end labeling; IF: immunofluorescence; WNT5A: Wnt family member 5A; MMP2: matrix metalloprotein; MMP9: matrix metalloprotein 9.

**Supplementary Figure 4** In vivo rescue assays were carried out. Hep3B cells were stably transfected with sh-NC, sh-PITPNA-AS1, + miR-876-5p inhibitor and sh-PITPNA-AS1#1 + miR-876-5p inhibitor + sh-WNT5A#1. (A) Images of tumors dissected from nude mice that were transplanted with different Hep3B cells. (B-C) Tumor volume and tumor weight in indicated groups were measured. (D) Protein expression of WNT5A, E-cadherin, N-cadherin, PCNA and Ki67 was evaluated with western blot assay after stable transfections. ^**^P<0.01 indicated statistically significant differences. PITPNA-AS1: phosphatidylinositol transfer protein alpha antisense RNA 1; WNT5A: Wnt family member 5A; PCNA: Proliferating Cell Nuclear Antigen.

**Table 1 Correlation between PITPNA-AS1 expression and clinical features of hepatocellular carcinoma patients (n = 60).** ^*^P<0.05, ^**^P<0.01 indicated statistically significant differences. PITPNA-AS1: phosphatidylinositol transfer protein alpha antisense RNA 1.
