# Supplementary figures and images for: PITPNA-AS1 abrogates the inhibition of miR-876-5p on WNT5A to facilitate hepatocellular carcinoma progression
Source: Cell Death Dis. 2019 Nov 7;10(11):844. doi: 10.1038/s41419-019-2067-2 (PMC6838072; doi:10.1038/s41419-019-2067-2)

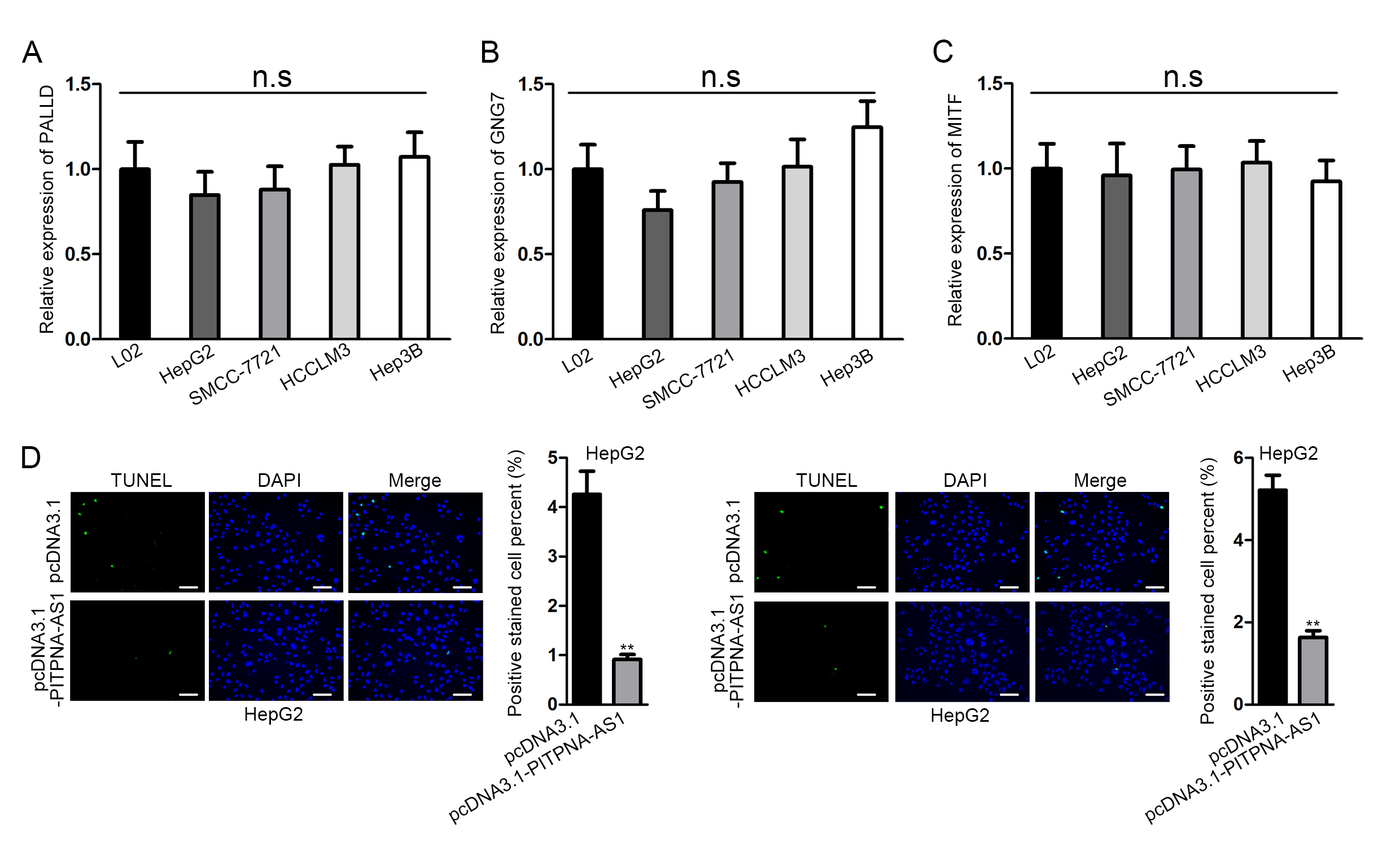

Supplement: Supplementary file 2 — Supplementary Figure 1 [file 41419_2019_2067_MOESM2_ESM.tif]

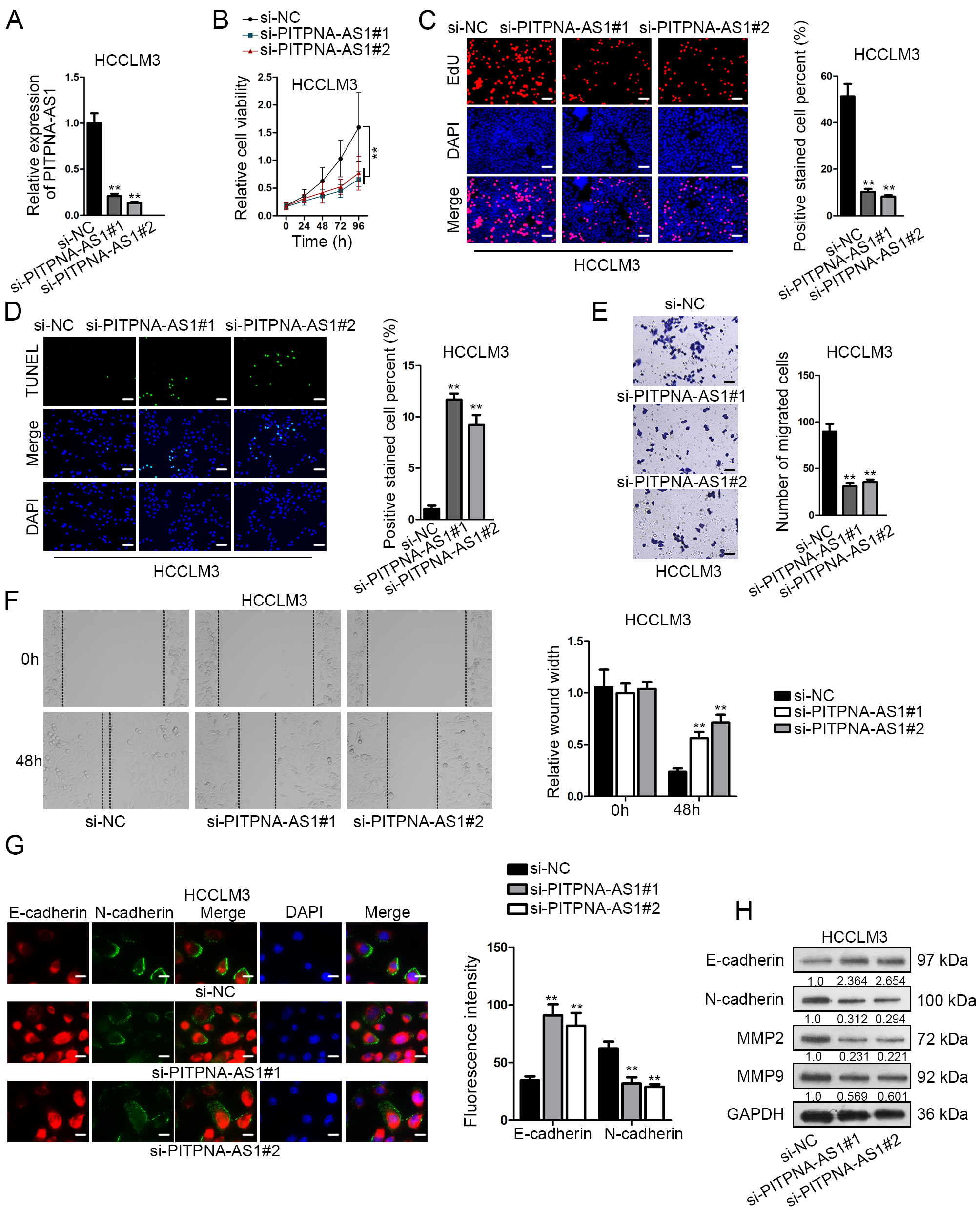

Supplement: Supplementary file 3 — Supplementary Figure 2 [file 41419_2019_2067_MOESM3_ESM.tif]

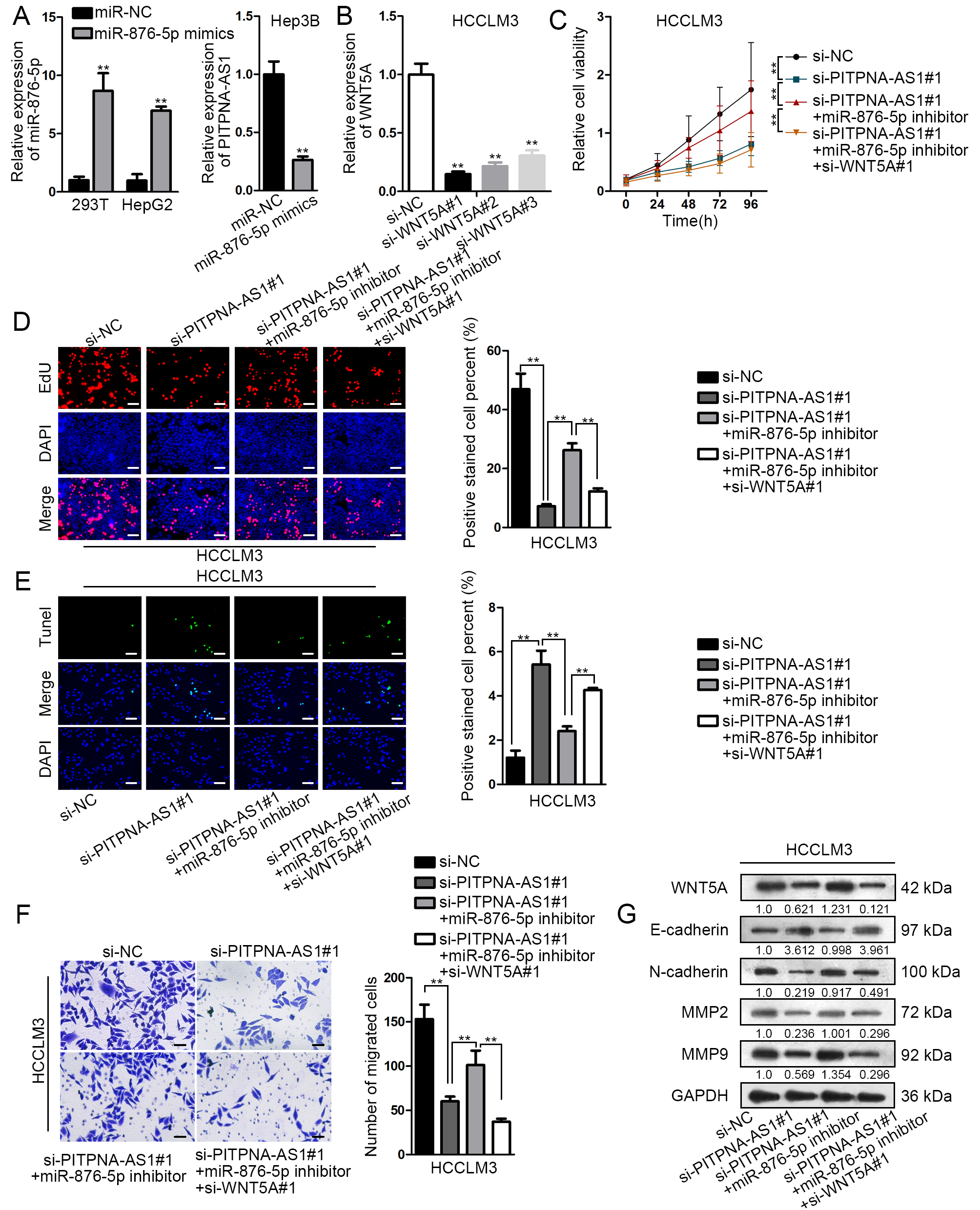

Supplement: Supplementary file 4 — Supplementary Figure 3 [file 41419_2019_2067_MOESM4_ESM.tif]

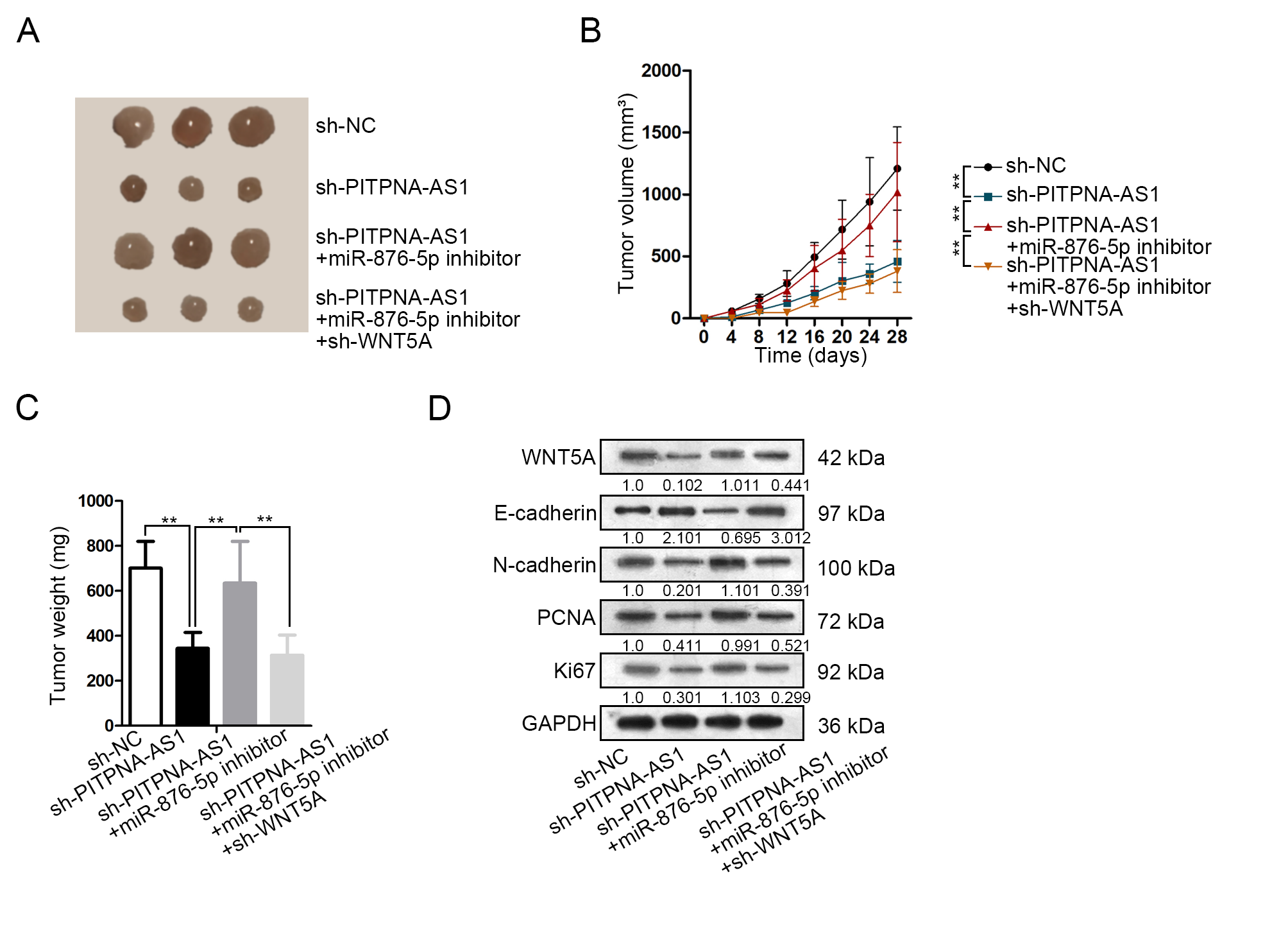

Supplement: Supplementary file 5 — Supplementary Figure 4 [file 41419_2019_2067_MOESM5_ESM.tif]
